# The Anti-Inflammatory Effects of Iberin on TNF-α-Stimulated Human Oral Epithelial Cells: In Vitro Research

**DOI:** 10.3390/biomedicines10123155

**Published:** 2022-12-06

**Authors:** Yoshitaka Hosokawa, Ikuko Hosokawa, Masahiro Shimoyama, Ayumi Fujii, Juri Sato, Kimitake Kadena, Kazumi Ozaki, Keiichi Hosaka

**Affiliations:** 1 Department of Regenerative Dental Medicine, Institute of Biomedical Sciences, Tokushima University Graduate School, Tokushima 770-8504, Tokushima, Japan; 2 Department of Oral Health Care Promotion, Institute of Biomedical Sciences, Tokushima University Graduate School, Tokushima 770-8504, Tokushima, Japan

**Keywords:** iberin, anti-inflammatory effect, oral epithelial cells

## Abstract

Iberin is a bioactive chemical found in cruciferous plants that has been demonstrated to have anticancer properties. However, there have been no reports on its effects on periodontal resident cells, and many questions remain unanswered. The aim of this study was to examine whether iberin had anti-inflammatory effects on human oral epithelial cells, including influences on signal transduction pathway activation in TNF-α-στιμυλατεd χελλσ. Iberin inhibited the production of interleukin (IL)-6 and C-X-C motif chemokine ligand 10 (CXCL10), as well as the expression of vascular cell adhesion molecule (VCAM)-1, inducible nitric oxide synthase (iNOS), and cyclooxygenase (COX)-2 in tumor necrosis factor (TNF)-α-stimulated TR146 cells, a human oral epithelial cell line. Moreover, iberin administration increased the expression of antioxidant signaling pathways, such as Heme Oxygenase (HO)-1 and NAD(P)H quinone dehydrogenase 1 (NQO1). Furthermore, we found that iberin could inhibit the activation of the nuclear factor (NF)-κB, signal transducer and activator of transcription (STAT)3, and p70S6 kinase (p70S6K)-S6 ribosomal protein (S6) pathways in TNF-α-stimulated TR146 cells. In conclusion, iberin reduced inflammatory mediator expression in human oral epithelial cells by preventing the activation of particular signal transduction pathways.

## 1. Introduction

Periodontitis is a chronic inflammatory disorder in periodontal tissues caused by periodontopathic bacteria such as *Porphyromonas gingivalis* [1]. An overactive immune system to microorganisms causes periodontal tissue destruction [1,2]. At the moment, periodontal lesions are treated with antibiotics that are supplied locally in the conventional treatment of periodontal disease. Since the problem of resistant bacteria has been raised [3], new anti-inflammatory compounds are expected to be identified.

Isothiocyanate is the generic name for substances with the chemical group –N=C=S, obtained by replacing the oxygen in the isocynate group with sulfur. Most natural isothiocyanates of plant origin are produced by enzymatic conversion of metabolites called glucosinolates [4]. Isothiocyanates are nutrients found mainly in cruciferous vegetables, and have been reported to have bioactive effects, including anticancer [5,6] and anti-inflammatory effects [6]. Iberin is an isothiocyanate that can be found in green and yellow vegetables, such as cruciferous plants. We selected iberin in this research because few reports have investigated the bioactive effects of iberin compared to the well-known isothiocianate, such as sulforaphane. Additionally, it has been reported that iberin is considered as a special member of the isothiocyanate family with little cytotoxicity to normal cells [7]. Several studies on the bioactive effects of iberin have been published in recent years [8,9]. Gong et al. reported that iberin treatment inhibits cell proliferation and induces cell apoptosis in oval cancer cell lines [8]. Pocasap et al. also demonstrated that iberin increases intracellular reactive oxygen species and tubulin depolymerization to have anticancer activity in hepatocellular carcinoma cell HepG2 cells [9]. However, there have been few studies looking at the anti-inflammatory properties of iberin. We also could not find any studies that have focused on the effects of iberin on periodontal resident cells.

We focused on the anti-inflammatory effects of iberin on human oral epithelial cell lines in this study. We specifically examined how iberin affected the expression of vascular cell adhesion molecule (VCAM)-1, inducible nitric oxide synthase (iNOS), and cyclooxygenase (COX-2), as well as the production of interleukin (IL)-6 and C-X-C motif chemokine ligand (CXCL)10 in tumor necrosis factor (TNF)-α-stimulated human oral epithelial cells. Iberin’s effects on the signal transducer and activator of transcription 3 (STAT3), nuclear factor (NF)-κB, and p70S6 kinase (p70S6K)-S6 ribosomal protein (S6) activation were also examined. Further, we investigated the effects of iberin on antioxidant expression, such as *Heme Oxygenase* (HO)-1 and NAD(P)H quinone dehydrogenase 1 (NQO1).

## 2. Materials and Methods

### 2.1. Cell Culture

Dr. Mark Herzberg (University of Minnesota, MN, USA) kindly provided the TR146 cell line, which is a human oral epithelial cell line. We used TR146 cells in this study. TR146 cells were cultured at 37 °C in humidified air with 5% CO_2_ in Ham’s F12 medium (Nakarai Tesque, Kyoto, Japan) supplemented with 10% fetal bovine serum (FBS) (JRH Biosciences, Lenexa, KS, USA), 1 mmol/L sodium pyruvate (Gibco, Grand Island, MI, USA), and antibiotics (penicillin G, 100 units/mL; streptomycin, 100 μg/mL; Gibco). When the cells reached 80% confluency, they were harvested for subculture with a 0.25% trypsin-ethylenediaminetetraacetic acid (EDTA) solution.

### 2.2. Cytotoxicity Assay

Cell viability was measured using Cell Count Reagent SF (Nakarai Tesque). TR146 cells were seeded in 96-well plates and incubated for 48 h. After 48 h, the media were discarded and 90 μL of Ham’s F12 medium with varying concentrations of iberin (Cayman Chemical, Ann Arbor, MI, USA) was added, and the cells were cultured for another 24 h. Then, we added 10 μL Cell Count Reagent SF and incubated the cells for 2 h before measuring the absorbance at 450 nm with a microplate reader.

### 2.3. IL-6 and CXCL10 Production in TR146 Cells

TR146 cells were stimulated for 24 h with TNF-α (100 ng/mL: Peprotech, Rocky Hill, NJ, USA) with or without iberin (1.875, 3.75, 7.5, or 15 μM). The concentration of IL-6 and CXCL10 in cell culture supernatant of TR146 cells were determined using DuoSet ELISA Development Systems (R&D systems, Minneapolis, MN, USA) in according to the manufacturer’s instructions.

### 2.4. Western Blot Analysis

TR146 cells were cultured in 12-well plates and total protein was collected in cell lysis buffer (Cell Signaling Technology, Danvers, MA, USA) after TNF-α (100 ng/mL) stimulation for 15, 30, or 60 min with or without iberin (7.5 or 15 μM) pretreatment for 1 h, or TNF-α (100 ng/mL) stimulation for 24 h with or without iberin (3.75, 7.5, or 15 μM). The BCA Protein Assay Kit (TaKaRa, Shiga, Japan) was used to determine the protein concentrations in the lysates. The same amount of protein was put onto a 4–20% SDS- polyacrylamide gel electrophoresis (PAGE) gel, which was then electrotransferred to a polyvinylidene difloride membrane. The membranes were blocked with 1% skim milk at room temperature for 1 h and then incubated with primary antibodies against VCAM-1 (Biolegend, San Diego, CA, USA), iNOS (Cell Signaling Technology), COX-2 (Cayman Chemical), HO-1 (Cell Signaling Technology), NQO1 (Cell Signaling Technology), Phospho-NF-κB p65 (Cell Signaling Technology), NF-κB p65 (Cell Signaling Technology), Phospho-IκB-α (Cell Signaling Technology), Phospho-STAT3 (Cell Signaling Technology), STAT3 (Cell Signaling Technology), Phospho-p70S6K (Cell Signaling Technology), p70S6K (Cell Signaling Technology), Phospho-S6 (Cell Signaling Technology), S6 (Cell Signaling Technology), or Glyceraldehyde-3-phosphate dehydrogenase (GAPDH) (Cell Signaling Technology). The membranes were then washed and treated with a corresponding horseradish peroxidase (HRP)-conjugated secondary antibody (Sigma-Aldrich, St. Louis, MO, USA). The ECL Prime Western-blotting detection system (Cytiva, Tokyo, Japan) was used to detect protein bands on X-ray films. The densities of bands of Western blot analysis were determined using Image J software (version 1.52p: NIH, Bethesda, MD, USA).

### 2.5. Statistical Analysis

One-way analysis of variance (ANOVA) was used to determine statistical significance, followed by a post hoc Tukey–Kramer test. *p* values less than 0.05 were regarded as significant.

## 3. Results

### 3.1. Effects of Iberin on Cell Viability of TR146 Cells

**Figure 1:**
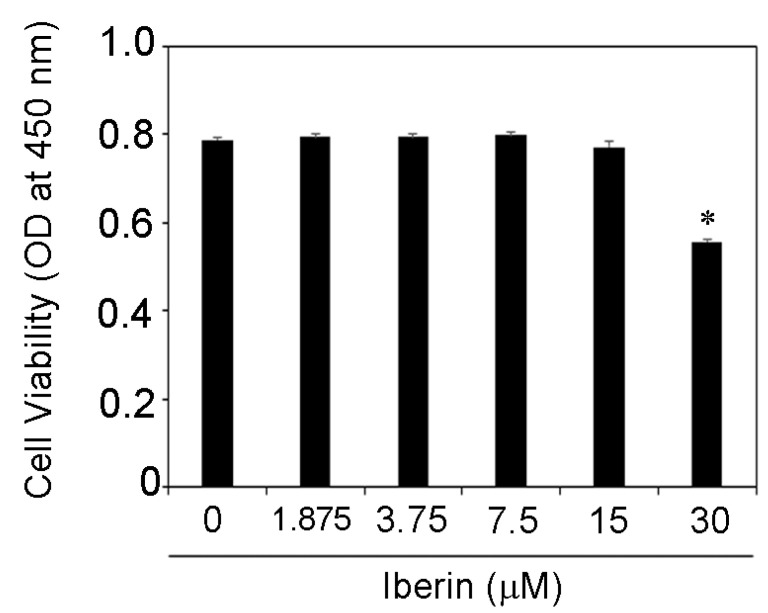
Effects of iberin on cell viability of TR146 cells. TR146 cells were seeded on 96-well cell culture plates, grown for two days, and then treated for 24 h with iberin (1.875–30 μM). The vitality of cells was determined using Cell Count Reagent SF. The data are presented as the mean SD of four independent experiments. * = *p* < 0.05, significantly different from TR146 cells not treated with iberin.

First, we investigated at how iberin affected the viability of TR146 cells. Figure 1 demonstrates that iberin (1.875–15 μM) had no effect on the viability of TR146 cells. As a result, iberin (1.875–15 μM) was used in this experiment.

### 3.2. Iberin Suppresses TNF-α-Induced IL-6 and CXCL10 Production in TR146 Cells

**Figure 2:**
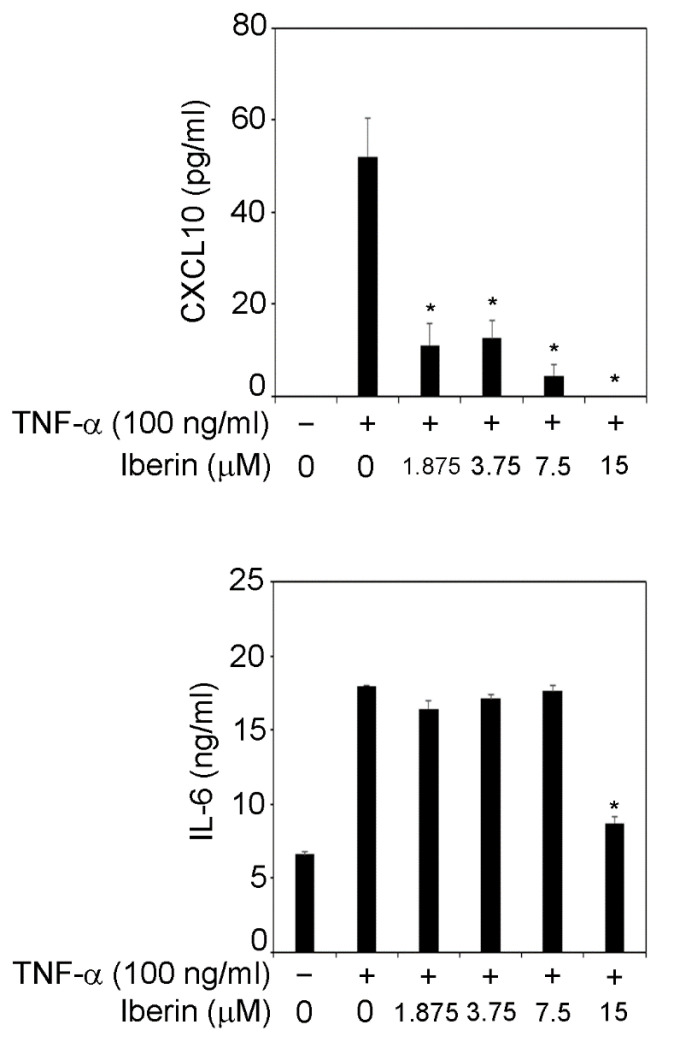
Effects of iberin on TNF-α-induced production of CXCL10 and IL-6. TR146 cells were grown for 24 h with TNF-α (100 ng/mL) with or without iberin (1.875–15 μM). CXCL10 and IL-6 levels in the supernatant were measured using the ELISA kits indicated in the Materials and Methods section. The data are presented as the mean SD of three independent experiments. * = *p* < 0.05, significantly different from TNF-α-stimulated TR146 cells without iberin.

IL-6 is a cytokine that is involved in osteoclast maturation and alveolar bone resorption in periodontal lesion [10]. CXCL10 can induce Th1 cell migration and accumulation in periodontal lesions, and Th1 cells are related to periodontal tissue destruction [11]. Therefore, we focused on IL-6 and CXCL10. TNF-α (100 ng/mL) increased IL-6 and CXCL10 release in TR146 cells, as shown in Figure 2. CXCL10 production in TNF-α-stimulated TR146 cells was significantly reduced when 1.875–15 μM iberin was added (Figure 2). Moreover, treatment with 15 μM iberin suppressed TNF-α-induced IL-6 production in TR146 cells (Figure 2).

### 3.3. Iberin Inhibits VCAM-1, iNOS, and COX-2 Expression in TNF-α-Stimulated TR146 Cells

**Figure 3:**
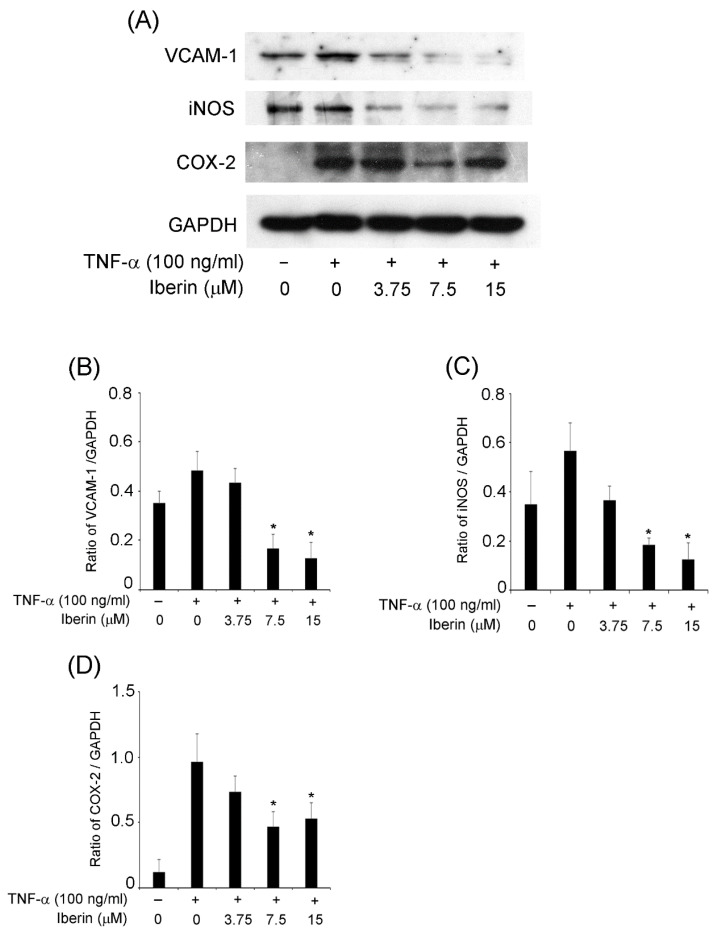
Effects of iberin on VCAM-1, iNOS, and COX-2 expression in TNF-α-stimulated TR146 cells. TR146 cells were pretreated for 1 h with iberin (3.75, 7.5, or 15 μM) before being stimulated with TNF-α (100 ng/mL). After 24 h of stimulation, the lysates were collected. Western blot analysis was used to investigate at the expression of VCAM-1, iNOS, and COX-2. (**A**) Representative Western blot image of the expression of VCAM-1, iNOS, COX-2, and GAPDH. (**B**–**D**) Quantification of protein expression by densitometry analysis of Western blots. Data are expressed as the mean ± SD of thre independent experiments. (* = *p* < 0.05 vs. TNF-α-stimulated TR146 cells without iberin).

Next, we wanted to know how iberin affects the expression of additional inflammatory mediators besides cytokines. We focused on VCAM-1, iNOS, and COX-2 expression since we knew they played a role in periodontal disease development [12,13,14]. Figure 3 demonstrates that iNOS expression was inhibited by 3.75–15 μM iberin, VCAM-1 expression also suppressed 7.5 and 15 μM iberin treatment, and 7.5 μM iberin clearly down-regulated COX-2 expression in TNF-α-stimulated TR146 cells.

### 3.4. Effects of Iberin on HO-1 and NQO1 Expression in TR146 Cells

**Figure 4:**
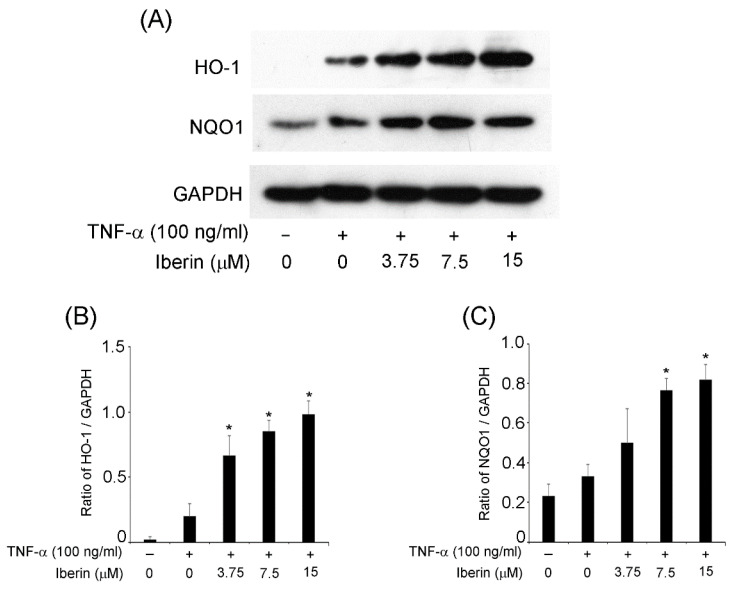
Effects of iberin on HO-1 and NQO1 expression in TNF-α-stimulated TR146 cells. TR146 cells were pretreated for 1 h with iberin (3.75, 7.5, or 15 μM) before being stimulated with TNF-α (100 ng/mL). After 24 h of stimulation, the lysates were collected. Western blot analysis was used to investigate at the expression of HO-1 and NQO1. (**A**) Representative Western blot image of the expression of HO-1, NQO-1, and GAPDH. (**B**,**C**) Quantification of protein expression by densitometry analysis of Western blots. Data are expressed as the mean ± SD of thre independent experiments. (* = *p* < 0.05 vs. TNF-α-stimulated TR146 cells without iberin).

Reactive oxygen species and the resulting oxidative stress are significant in the onset and progression of periodontal disease [15]. As a result, it is critical to enhance the expression of the antioxidants (HO-1 and NQO1) in periodontal lesions. Thus, we looked into the effects of iberin on HO-1 and NQO1 expression. Figure 4 indicates that iberin treatment significantly increased the expression of HO-1 and NQO1 in TR146 cells compared to TR146 cells that were not treated with iberin.

### 3.5. Effects of Iberin on NF-κB, STAT3, and p70S6K-S6 Signaling Pathways in TNF-α-Stimulated TR146 Cells

**Figure 5:**
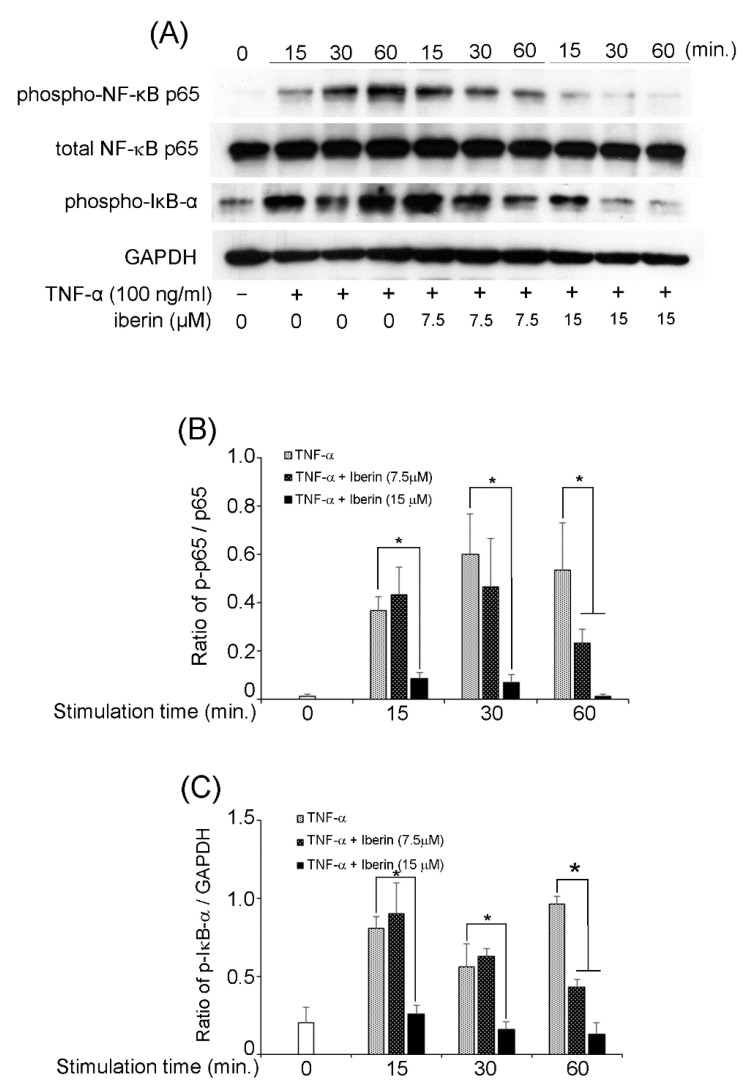
The effects of iberin treatment on the activation a NF-κB pathway in TNF-α stimulated TR146 cells. TR146 cells were pretreated with iberin (7.5 or 15 μM) for 1 h before being stimulated with TNF-α for 15, 30, or 60 min, and the phosphorylation of NF-κB p65 and IκB-α was measured using Western immunoblotting. (**A**) Representative Western blot image of the expression of phospho-NF-κB p65, total NF-κB p65, phospho-IκB-α, and GAPDH. (**B**,**C**) Quantification of protein expression by densitometry analysis of Western blots. Data are expressed as the mean ± SD of three independent experiments. (* = *p* < 0.05).

**Figure 6:**
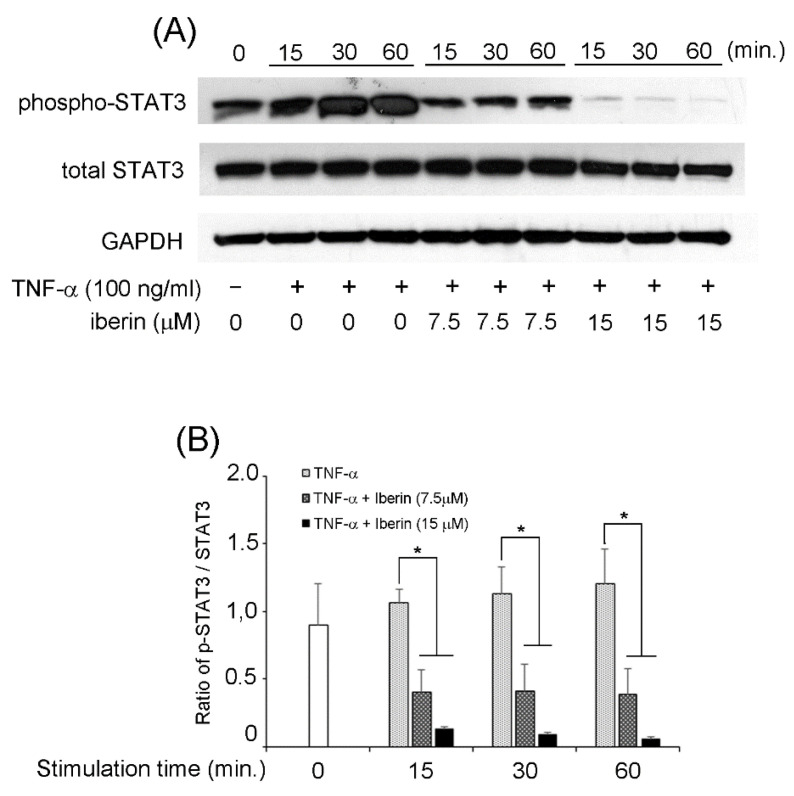
The effect of iberin treatment on the activation of a STAT3 pathway in TNF-α stimulated TR146 cells. TR146 cells were pretreated with iberin (7.5 or 15 μM) for 1 h before being stimulated with TNF-α for 15, 30, or 60 min, and the phosphorylation of STAT3 was evaluated using Western immunoblotting. (**A**) Representative Western blot image of the expression of phospho-STAT3, total STAT3, and GAPDH. (**B**) Quantification of protein expression by densitometry analysis of Western blots. Data are expressed as the mean ± SD of three independent experiments. (* = *p* < 0.05).

**Figure 7:**
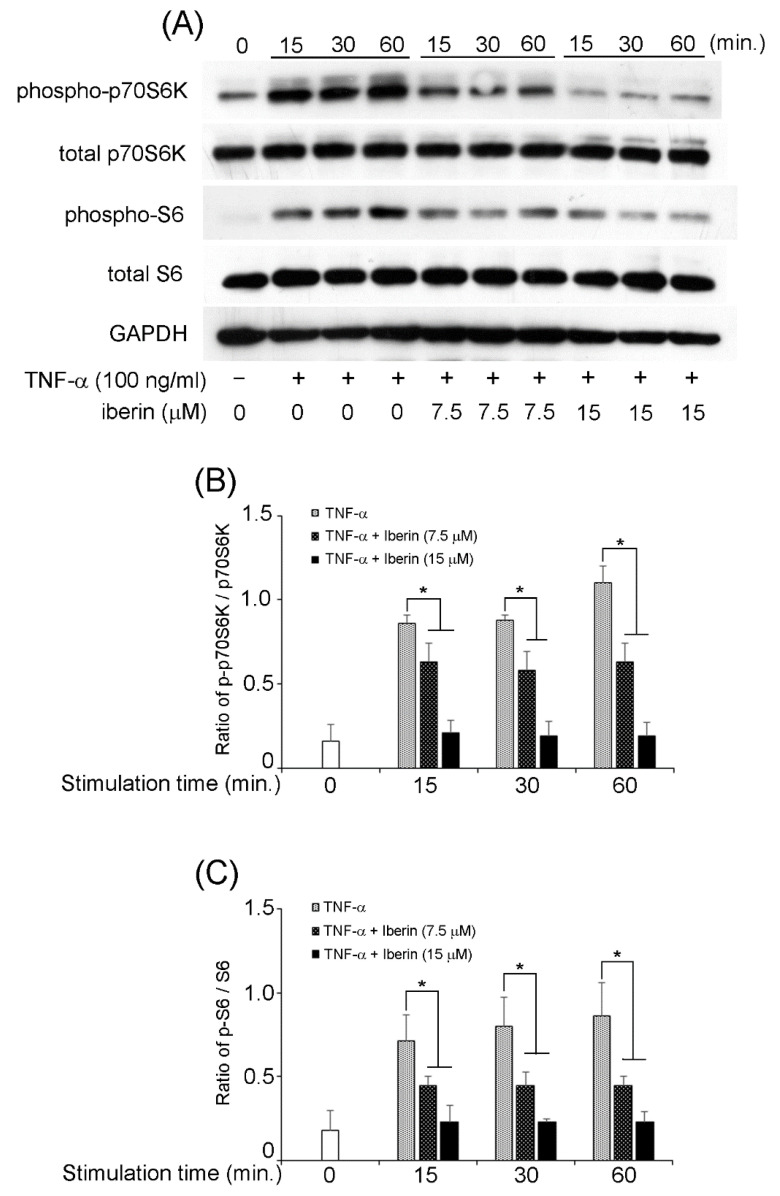
The effect of iberin treatment on the activation of a p70S6K-S6 pathway in TNF-α stimulated TR146 cells. TR146 cells were pretreated with iberin (7.5 or 15 μM) for 1 h before being stimulated with TNF-α for 15, 30, or 60 min, and the phosphorylation of p70S6K and S6 was measured using Western immunoblotting. (**A**) Representative Western blot image of the expression of phospho-p70S6K, total p70S6K, phospho-S6, total S6, and GAPDH. (**B**,**C**) Quantification of protein expression by densitometry analysis of Western blots. Data are expressed as the mean ± SD of thre independent experiments. (* = *p* < 0.05).

We previously reported that TNF-α could activate NF-κB, STAT3, and p70S6K-S6 pathways in TR146 cells [16]. Therefore, we decided to investigate the effects of iberin on the activation of the mentioned signaling pathways. In TR146 cells, 15 μM iberin reduced TNF-α-induced phosphorylation of NF-κB p65 and IκB-α (Figure 5). Figure 6 demonstrates that 7.5 and 15 μM iberin administration significantly reduced STAT3 phosphorylation. Treatment with 7.5 or 15 μM iberin effectively reduced the levels of p70S6K and S6 phosphorylation in TNF-α-stimulated TR146 cells (Figure 7). These findings explain how iberin pretreatment inhibited various signaling pathways in TR146 cells, including NF-κB, STAT3, and p70S6K-S6.

## 4. Discussion

Periodontitis is a chronic inflammatory disease caused by periodontopathogenic bacteria [1]. Local administration of antimicrobial drugs has been used for the treatment of periodontitis, but due to the problem of bacterial resistance, the discovery of new bioactive compounds with anti-inflammatory action has lately been requested [3]. We concentrated on the bioactive compounds found in cruciferous plants, a popular food worldwide. This is because we felt that all people could utilize it securely.

We were able to find a few articles that investigated the anti-cancer properties of iberin [8,9]. However, there have been limited studies on the anti-inflammatory properties of iberin. Shibata et al. discovered that iberin had an anti-inflammatory effect on toll-like receptor (TLR) ligand-stimulated RAW 264.7 cells, a macrophage cell line. In lipopolysaccharide (LPS) or Pam3CSK4-treated RAW 264.7 cells, iberin inhibited iNOS, COX-2, TNF-α, and IL-1α expression, as well as NF-κB activation. They also reported that because iberin inhibited TLR dimerization, it did not reduce the NF-κB activity generated by phorbol 12-myristate 13-acetate, a TLR-independent proinflammatory ligand [17]. Although we did not use TLR ligands in this investigation, iberin decreased the expression of inflammatory mediators. We need to conduct further research to figure out how iberin decreased TNF-α-induced inflammatory mediator production.

Many studies have revealed that isothiocyanates, such as sulforaphane, activate NF-E2-related factor(Nrf)2/HO-1 signaling [18,19,20,21]. However, there have been few reports that iberin stimulates antioxidants such as HO-1 and NQO1. Ernst et al. showed that iberin administration raised nuclear Nrf2 levels and HO-1 expression in the murine fibroblast cell line NIH3T3 [22]. We believe this is the second article showing that iberin can produce antioxidants.

In TNF-α-stimulated TR146 cells, iberin inhibited the activation of NF-κB, STAT3, and p70S6K-S6. Our prior research also found that 6-MSITC, an isothiocyanate containing wasabi, inhibited the NF-κB, STAT3, and p70S6K-S6 pathways [16]. Shibata et al. reported NF-κB activation was inhibited by iberin treatment in LPS-stimulated macrophages [17]. Recently, it was reported that STAT3 phosphorylation in immortalized bone marrow-derived macrophages stimulated by LPS is inhibited by sulforaphane, a type of isothiocyanate [23]. It has also been reported that sulforaphane inhibits p70S6K phosphorylation in platelet-derived growth factor-treated vascular smooth muscle cells [24]. Although further studies are needed, based on our findings and those of others, suppression of the NF-κB, STAT3, and p70S6K-S6 pathways may be a common property of isothiocyanates.

Recently, it has been reported that the bioactive effects of isothiocyanates occur via bitter taste receptors (T2R). Qin et al. reported that N-C=S-containing compounds including isothiocyanates are detected via T2R38 receptors in Caco-2 cells [25]. It has also been shown that T2R is expressed in oral epithelial cells and is involved in the innate immune response. Previous reports showed that T2R14 is expressed in gingival epithelial cells (GECs) and interacts with competence-stimulating peptides (CSPs) secreted by the *Storeptococcus mutans* [26,27]. T2R agonists induce TNF-α, IL-6, IL-8 and beta-defensin2 production in GECs [27,28]. These reports indicate that in the oral cavity, taste receptors are actively involved in the innate response by recognizing bacterial components [29]. In addition, isothiocyanates such as iberin may bind to bitter taste receptors in the oral cavity and exert bioactive effects. We believe that more research on the regulation of inflammation in the oral cavity with a focus on bitter taste receptors is needed.

## 5. Conclusions

In this paper, we have shown that iberin can decrease the production of inflammatory mediators such as IL-6, CXCL10, VCAM-1, iNOS, and COX-2 while increasing antioxidants in TNF-α-treated human oral epithelial cells. Iberin administration to the periodontal legions may therefore be utilized to treat periodontitis. However, in order to use iberin for the treatment of periodontitis in humans, it is necessary to study its effects on periodontal tissue constituent cells other than human oral epithelial cells. It is also important to examine the effects of iberin on animal models of periodontitis.

## Data Availability

Not applicable.

## References

[1] Darveau R.P. (2010). Periodontitis: A polymicrobial disruption of host homeostasis. Nat. Rev. Microbiol..

[2] Huang J., Cai X., Ou Y., Zhou Y., Wang Y. (2018). Resolution of inflammation in periodontitis: A review. Int. J. Clin. Exp. Pathol..

[3] Mahuli S.A., Zorair A.M., Jafer M.A., Sultan A., Sarode G., Baeshen H.A., Raj A.T., Sarode S., Patil S. (2020). Antibiotics for Periodontal Infections: Biological and Clinical Perspectives. J. Contemp. Dent. Pract..

[4] Zhang Y. (2012). The molecular basis that unifies the metabolism, cellular uptake and chemopreventive activities of dietary isothiocyanates. Carcinogenesis.

[5] Zhang Y., Huang H., Jin L., Lin S. (2022). Anticarcinogenic Effects of Isothiocyanates on Hepatocellular Carcinoma. Int. J. Mol. Sci..

[6] Tarar A., Peng S., Cheema S., Peng C.A. (2022). Anticancer Activity, Mechanism, and Delivery of Allyl Isothiocyanate. Bioengineering.

[7] Jadhav U., Ezhilarasan R., Vaughn S.F., Berhow M.A., Mohanam S. (2007). Iberin induces cell cycle arrest and apoptosis in human neuroblastoma cells. Int. J. Mol. Med..

[8] Gong T.T., Guo Q., Li X., Zhang T.N., Liu F.H., He X.H., Lin B., Wu Q.J. (2021). Isothiocyanate Iberin inhibits cell proliferation and induces cell apoptosis in the progression of ovarian cancer by mediating ROS accumulation and GPX1 expression. Biomed. Pharmacother..

[9] Pocasap P., Weerapreeyakul N., Thumanu K. (2019). Alyssin and Iberin in Cruciferous Vegetables Exert Anticancer Activity in HepG2 by Increasing Intracellular Reactive Oxygen Species and Tubulin Depolymerization. Biomol. Ther..

[10] Suda T., Udagawa N., Nakamura I., Miyaura C., Takahashi N. (1995). Modulation of osteoclast differentiation by local factors. Bone.

[11] Cavalla F., Letra A., Silva R.M., Garlet G.P. (2021). Determinants of Periodontal/Periapical Lesion Stability and Progression. J. Dent. Res..

[12] Crawford J.M., Watanab K. (1994). Cell adhesion molecules in inflammation and immunity: Relevance to periodontal diseases. Crit. Rev. Oral Biol. Med..

[13] Uğar-Cankal D., Ozmeric N. (2006). A multifaceted molecule, nitric oxide in oral and periodontal diseases. Clin. Chim. Acta.

[14] Fracon R.N., Teófilo J.M., Satin R.B., Lamano T. (2008). Prostaglandins and bone: Potential risks and benefits related to the use of nonsteroidal anti-inflammatory drugs in clinical dentistry. J. Oral Sci..

[15] Paul O., Arora P., Mayer M., Chatterjee S. (2021). Inflammation in Periodontal Disease: Possible Link to Vascular Disease. Front. Physiol..

[16] Shimoyama M., Hosokawa Y., Hosokawa I., Ozaki K., Hosaka K. (2022). 6-(Methylsulfinyl) Hexyl Isothiocyanate Inhibits IL-6 and CXCL10 Production in TNF-α-Stimulated Human Oral Epithelial Cells. Curr. Issues Mol. Biol..

[17] Shibata T., Nakashima F., Honda K., Lu Y.J., Kondo T., Ushida Y., Aizawa K., Suganuma H., Oe S., Tanaka H. (2014). Toll-like receptors as a target of food-derived anti-inflammatory compounds. J. Biol. Chem..

[18] Thiruvengadam M., Venkidasamy B., Subramanian U., Samynathan R., Ali Shariati M., Rebezov M., Girish S., Thangavel S., Dhanapal A.R., Fedoseeva N. (2021). Bioactive Compounds in Oxidative Stress-Mediated Diseases: Targeting the NRF2/ARE Signaling Pathway and Epigenetic Regulation. Antioxidants.

[19] Wei J., Zhao Q., Zhang Y., Shi W., Wang H., Zheng Z., Meng L., Xin Y., Jiang X. (2021). Sulforaphane-Mediated Nrf2 Activation Prevents Radiation-Induced Skin Injury through Inhibiting the Oxidative-Stress-Activated DNA Damage and NLRP3 Inflammasome. Antioxidants.

[20] Ruhee R.T., Ma S., Suzuki K. (2020). Protective Effects of Sulforaphane on Exercise-Induced Organ Damage via Inducing Antioxidant Defense Responses. Antioxidants.

[21] Ruhee R.T., Ma S., Suzuki K. (2019). Sulforaphane Protects Cells against Lipopolysaccharide-Stimulated Inflammation in Murine Macrophages. Antioxidants.

[22] Ernst I.M., Palani K., Esatbeyoglu T., Schwarz K., Rimbach G. (2013). Synthesis and Nrf2-inducing activity of the isothiocyanates iberverin, iberin and cheirolin. Pharmacol. Res..

[23] Bahiraii S., Brenner M., Yan F., Weckwerth W., Heiss E.H. (2022). Sulforaphane diminishes moonlighting of pyruvate kinase M2 and interleukin 1β expression in M1 (LPS) macrophages. Front. Immunol..

[24] Shawky N.M., Segar L. (2017). Sulforaphane inhibits platelet-derived growth factor-induced vascular smooth muscle cell proliferation by targeting mTOR/p70S6kinase signaling independent of Nrf2 activation. Pharmacol. Res..

[25] Qin C., Qin Z., Zhao D., Pan Y., Zhuang L., Wan H., Di Pizio A., Malach E., Niv M.Y., Huang L. (2019). A bioinspired in vitro bioelectronic tongue with human T2R38 receptor for high-specificity detection of N-C=S-containing compounds. Talanta.

[26] Gil S., Coldwell S., Drury J.L., Arroyo F., Phi T., Saadat S., Kwong D., Chung W.O. (2015). Genotype-specific regulation of oral innate immunity by T2R38 taste receptor. Mol. Immunol..

[27] Medapati M.R., Singh N., Bhagirath A.Y., Duan K., Triggs-Raine B., Batista E.L., Chelikani P. (2021). Bitter taste receptor T2R14 detects quorum sensing molecules from cariogenic *Streptococcus mutans* and mediates innate immune responses in gingival epithelial cells. FASEB J..

[28] Medapati M.R., Bhagirath A.Y., Singh N., Schroth R.J., Bhullar R.P., Duan K., Chelikani P. (2021). Bitter Taste Receptor T2R14 Modulates Gram-Positive Bacterial Internalization and Survival in Gingival Epithelial Cells. Int. J. Mol. Sci..

[29] Xi R., Zheng X., Tizzano M.J. (2022). Role of Taste Receptors in Innate Immunity and Oral Health. J. Dent. Res..

